# Results of weekday-on and weekend-off administration schedule of sunitinib therapy for advanced renal cell carcinoma

**DOI:** 10.1007/s10147-018-1332-1

**Published:** 2018-08-09

**Authors:** Atsunari Kawashima, Motohide Uemura, Taigo Kato, Takeshi Ujike, Akira Nagahara, Kazutoshi Fujita, Ryoichi Imamura, Yohei Yamanaka, Eisuke Tomiyama, Go Tanigawa, Yasushi Miyagawa, Toshiaki Yoshioka, Osamu Miyake, Norio Nonomura

**Affiliations:** 1 Department of Urology, Graduate School of Medicine, Osaka University, 2-2 Yamadaoka, Suita, Osaka 5650871 Japan; 2 Department of Therapeutic Urologic Oncology, Graduate School of Medicine, Osaka University, 2-2 Yamadaoka, Suita, Osaka 5650871 Japan; 3 Department of Urology, Osaka Police Hospital, Osaka, Japan; 4 Department of Urology, Osaka General Medical Center Hospital, Osaka, Japan; 5 Department of Urology, Sumitomo Hospital, Osaka, Japan; 6 Department of Urology, Toyonaka Municipal Hospital, Toyonaka, Osaka Japan

**Keywords:** Alternative schedule, Relative dose intensity, Molecular targeted therapy, Renal cell carcinoma, Sunitinib, Weekday-on, Weekend-off

## Abstract

**Background:**

Sunitinib is widely prescribed as first-line therapy for metastatic renal cell carcinoma. To reduce the ratio of severe adverse events and improve the relative dose intensity, we prospectively tried our own alternative medication schedule, which we called the “weekday-on and weekend-off regimen”. Here we report the results of this regimen compared to the conventional medication schedule.

**Methods:**

In total, 58 patients were enrolled in this study. Twenty patients were treated under the alternative schedule (group I: weekday-on and weekend-off regimen) and 38 patients were treated using the conventional schedule (group II: 4 weeks on and 2 weeks off regimen). The relative dose intensity (6W-RDI) and prognoses were compared between the two groups.

**Results:**

Median 6W-RDI of all the patients was 75.0%. Group I patients demonstrated significantly higher 6W-RDI compared to group II (77.2 vs. 70.4%) (*p* = 0.019). Multivariate analysis showed that the alternative sunitinib administration schedule was significantly associated with maintaining 6W-RDI above 75% for RCC patients treated with sunitinib (OR 3.592, 95% CI 1.042–12.383, *p* = 0.043). On the other hand, there were no significant differences between 2 groups regarding occurrence rate of severe adverse events and prognosis by multivariate analysis.

**Conclusions:**

We report the results of an alternative medication schedule, the “weekday-on and weekend-off regimen”, as a means of increasing 6W-RDI for metastatic RCC patients.

## Introduction

Since several oral receptor tyrosine kinase inhibitors (TKIs) and the mechanistic target of rapamycin (mTOR) inhibitors were approved for metastatic renal cell carcinoma (mRCC), sunitinib is widely prescribed as the first-line therapy for mRCC patients along with pazopanib, [1, 2] and the prognosis of mRCC patients has improved compared to the era of cytokine therapy [3, 4]. In the real-world setting, both global and Japanese case registration studies allowed for certain therapeutic effects and prolongation of progression-free survival and overall survival time [5, 6]. Yet in addition to its therapeutic effect, treatment-related severe adverse events (AEs) appeared in some patients, leading to dose reduction and in some cases discontinuation of drug administration. Previously, we reported that maintenance of relative dose intensity (RDI) during the first course of treatment is important to improve the prognosis for mRCC patients treated by oral TKIs such as sunitinib [7, 8]. To reduce the ratio of severe adverse events and get higher RDI, three alternative medication schedules for sunitinib therapy (continuous one-daily dosing regimen [9–11], 2 weeks on and 1 week off regimen [12–14] and weekday-on and weekend-off regimen [15]) have been pursued, including some prospective studies.

We prospectively tried to establish our own alternative medication schedule, which we called the “weekday-on and weekend-off regimen”. Here we report the results of our prospective study and compare this regimen to the conventional medication schedule.

## Patients and methods

### Patients enrolled in this study

**Fig. 1:**
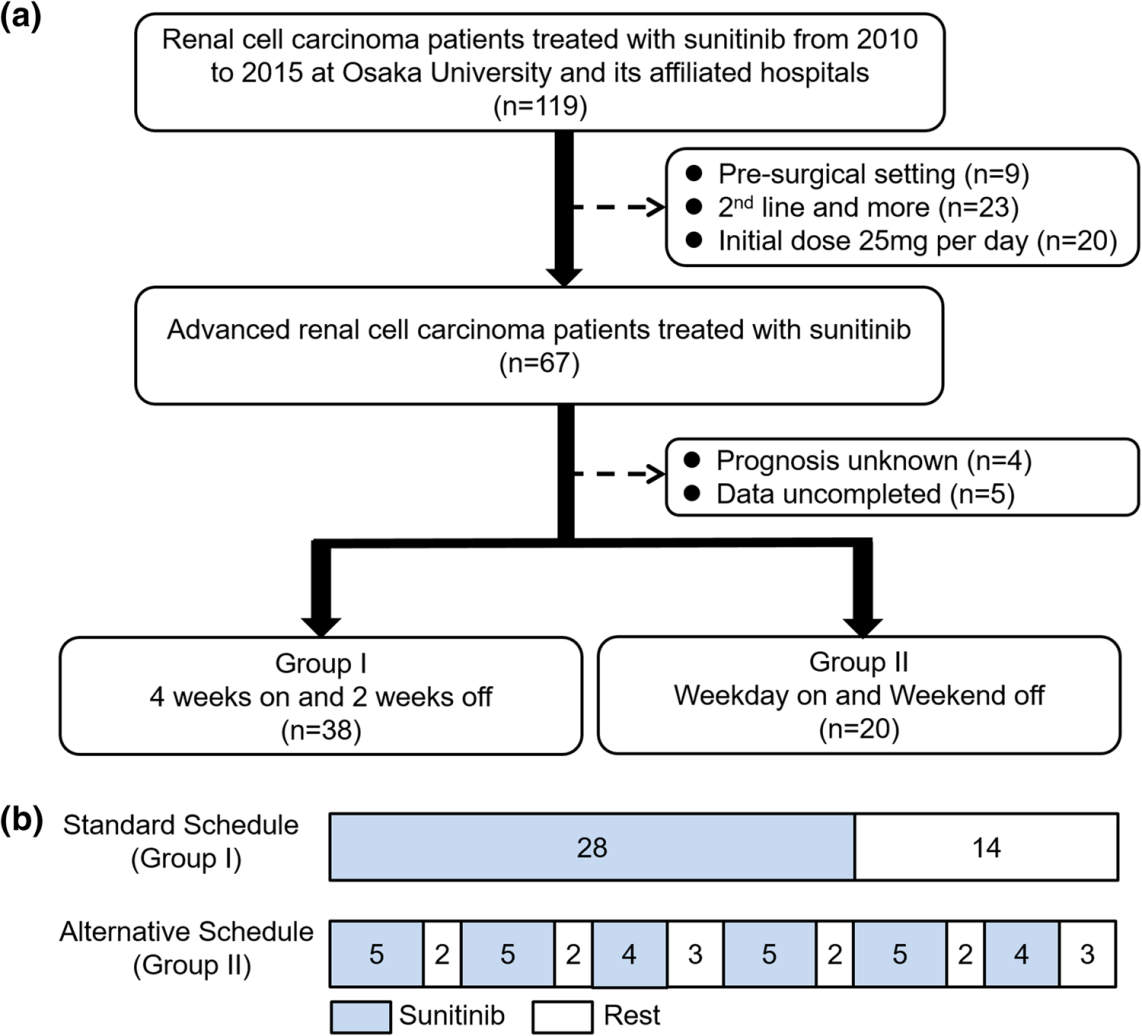
**a** Criteria of the patient selection enrolled in this study. **b** Conventional schedule and alternative schedule. The numbers in the box represent the number of days

One hundred and nineteen RCC patients treated with sunitinib from 2010 to 2015 at Osaka University and its affiliated hospitals were collected in this study (Fig. 1a). In total, 52 patients were excluded due to pre-surgical setting (*n* = 9), 2nd line and more setting (*n* = 23), the usage of initial dose of 25 mg per day (*n* = 20), prognosis unknown (*n* = 4) and data uncompleted (*n* = 5). Of 67 patients, 38 patients were treated under the conventional schedule (4 weeks on and 2 weeks off regimen: group I) and 20 patients were treated under the alternative schedule (weekday-on and weekend-off regimen: group II). The initially diagnosed tumours were staged according to the 7th American Joint Committee on Cancer staging classification [16]. The patient characteristics including laboratory findings were evaluated just before being treated with sunitinib medication. Clinical laboratory data collected for analysis included serum sodium concentration, estimated glomerular filtration ratio (eGFR) and C-reactive protein (CRP).

### Alternative administration schedule

Under our alternative schedule, sunitinib was administered only on 5 consecutive days for 2 weeks and 3 consecutive days for 1 week. The same dose intensity of conventional schedule was secured under the alternative schedule every 6 weeks as one course (Fig. 1b). Relative dose intensity for the first 6 weeks (6 weeks-RDI) was calculated as previously reported [7, 8]. Maximum RDI of general schedule and alternative schedule was 100%. This alternative schedule was approved by institutional review board of all the participating institutions, and was registered with the Japanese University Hospital Medical Information Network clinical trial center (ID: UMIN000011649).

Selection of conventional schedule or alternative schedule was left to the judgement of each physician. Dose reductions and/or discontinuation were also applied in cases of disease progression, unacceptable toxicity or by decision of the physicians. Resuming the sunitinib medication was also left to decision of the physicians when medication was discontinued due to severe AEs or patient’s preference.

### Follow-up regimens

Patient’s follow-up generally consisted of history, physical examination, routine blood work, abdominopelvic computed tomography (CT), and chest radiography. Elective bone scan and chest CT were performed when clinically indicated by several urologists. Tumor response was evaluated by the treating urologist every 1–3 months according to the Response Evaluation Criteria in Solid Tumors (RECIST) guidelines [17]. The AEs related to sunitinib therapy were recorded according to the National Cancer Institute’s Common Terminology Criteria for Adverse Events version 4.0 [18].

### Statistical analysis

The main objectives of this study were to compare the amount of 6W-RDI and the incidence of AEs between conventional and alternative schedule. Comparisons between the amount of 6W-RDI and clinical feature including administration schedule were evaluated by Mann–Whitney *U* test and logistic regression analysis. Progression-free survival (PFS) time was measured from the date of initiation of sunitinib therapy until documented disease progression, death from disease progression, or the date of the patient’s last follow-up visit. Distributions of PFS times were estimated with the Kaplan–Meier method, and associations between PFS and the clinical items were assessed with the log-rank test. As a multivariate analysis, Cox regression analysis using a step-wise forward selection with *p* < 0.1 as the criterion for model entry or stay was used. Statistical analysis was performed with the Statistical Package for the Social Sciences software, version 20.0 (SPSS, Inc., Chicago, IL). A value of *p* < 0.05 was considered statistically significant.

## Results

### Patient characteristics

Clinical characteristics of the 58 patients are described in Table 1. Median patient age was 66 (range 43–85) years, and 47 patients (81.0%) were male. ECOG performance status of all the patients was 0 or 1. The initial sunitinib dose was 50 mg for 33 patients (56.9%) and 37.5 mg for the others. The initial dose was stratified by body surface area (50 mg/day ≥ 1.6 m^2^, 37.5 mg/day < 1.6 m^2^). Twenty-two patients (37.9%) had hyponatremia (137 mEq/L and less). Estimated GFR of 23 patients (39.7%) was less than 44 mL/min/1.72 m^2^, which was defined as stage 3b and more of chronic kidney disease. CRP of 19 patients (32.8%) was 1 mg/dL and more.

**Table 1:** Characteristics of patients enrolled in this study

	Total (*n* = 58)	Group I (*n* = 38)	Group II (*n* = 20)	*p*
Age (median)	43–85 (66)	43–80 (67.5)	44–85 (64.5)	0.636
Gender				
Male	47	33	14	0.163
Female	11	5	6	
Initial sunitinib dose				
50 mg	33	23	10	0.578
37.5 mg	25	15	10	
Serum sodium concentration				
≤ 137 mEq/L	22	13	9	0.570
≥ 138 mEq/L	36	25	11	
Estimated GFR				
< 44 mL/min/1.73 m^2^	23	15	8	0.969
≥ 44 mL /min/1.73 m^2^	35	23	12	
C-reactive protein				
< 1 mg/dL	39	28	11	0.239
≥ 1 mg/dl	19	10	9	
Discontinuation of sunitinib within follow-up time	14	9	5	0.911
Relative dose intensity for initial 6 weeks (%) (raw data)	27–100 (75.0)	27–100 (70.4)	38.0–100 (77.2)	0.019

*ECOG* Eastern Cooperative Oncology Group, *GFR* glomerular filtration rate

### Relative dose intensity for initial 6 weeks according to the schedule of sunitinib administration

**Fig. 2:**
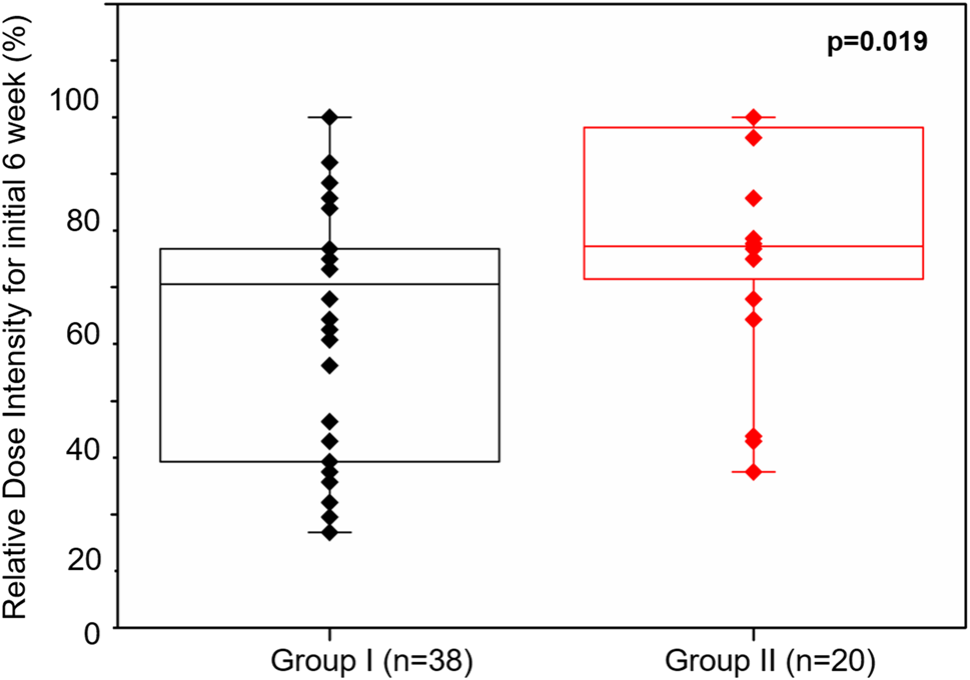
Relative dose intensity for initial 6 weeks according to the schedule of sunitinib administration. Statistical analysis was performed by Mann–Whitney’s test. Group I: 4 weeks on/2 weeks off, Group II: weekday-on/weekend-off

Median 6W-RDI of all the patients was 75.0% (27–100%). In the “4 week on and 2 week off regimen” group, median 6W-RDI was 70.4% (27–100%), whereas in the “weekday-on and weekend-off regimen” group, median 6W-RDI was 77.2% (38–100%). The 6W-RDI of group II was significantly higher than that of group I (*p* = 0.019) (Fig. 2).

Overall, 33 patients (56.9%) achieved 75% and more 6W-RDI. Fifteen patients (75.0%) in group I and 18 patients (47.4%) in group II were able to achieve 75% and more 6W-RDI. Statistically, only the alternative sunitinib administration schedule was significantly correlated [odds ratio (OR) 3.333, 95% confidence interval (CI) 1.008–11.020, *p* = 0.048]. In multivariate analysis, the alternative sunitinib administration schedule was shown to be an important clinical feature for maintaining high 6W-RDI for RCC patients treated by sunitinib (OR 3.592, 95% CI 1.042–12.383, *p* = 0.043) (Table 2).

**Table 2:** Result of univariate and multivariate analysis about the clinical features significantly correlated with achievement of relative dose intensity more than 75%. Statistical analysis was performed by logistic regression analysis

Clinical features	Univariate analysis			Multivariate analysis		
	OR	95% CI	*p*	OR	95% CI	*p*
Age (median) (< 65 vs. ≥ 65)	0.943	0.331–2.682	0.912			
Gender (male vs. female)	0.889	0.237–3.328	0.861			
Initial sunitinib dose (50 vs. 37.5 mg)	0.527	0.183–1.519	0.236			
Sunitinib administration schedule (Group I vs. Group II)	3.333	1.008–11.020	**0.048**	3.592	1.042–12.383	**0.043**
Serum sodium concentration (≤ 137 mEq/l vs. ≥ 138 mEq/l)	0.467	0.154–1.416	0.178			
Estimated GFR (< 44 mL/min/1.73 m2 vs. ≥ 44 mL/min/1.73 m^2^)	2.492	0.846–7.338	0.098	2.713	0.871–8.451	0.085
C-reactive protein (< 1 mg/dL vs. ≥ 1 mg/dL)	1.062	0.350–3.221	0.915			

*OR* odds ratio, *CI* confidence interval, *GFR* glomerular filtration rate

### Severe adverse events (Grade 3 and 4) stratified by administration schedule

In total, nine patients (15.5%) suffered from severe (Grade 3 or 4) AEs within one course (Table 3). There were no patients with Grade 5 AEs. Six patients (15.8%) in group I and 1 patient (5.0%) in group II had severe thrombocytopenia. Occurrence rate of group I was higher than that of group II although the difference was not significant (*p* = 0.403). Severe liver dysfunction, hypertension, and hand-foot skin reaction occurred in each one patient of group II. In addition, general fatigue has occurred in one patient of group I.

**Table 3:** Severe adverse events (Grade 3 and 4) stratified by administration schedule

Adverse events (Grade 3 or 4)	Group I (n = 38)	Group II (n = 20)	*p*
Thrombocytopenia	6 (15.8%)	1 (5.0%)	0.403
Liver dysfunction	0	1 (5.0%)	0.345
Hypertension	0	1 (5.0%)	0.345
Hand-foot skin reaction	0	1 (5.0%)	0.345
General fatigue	1 (2.6%)	0	1.000

### PFS of total cases and stratified by some clinical characteristics

**Fig. 3:**
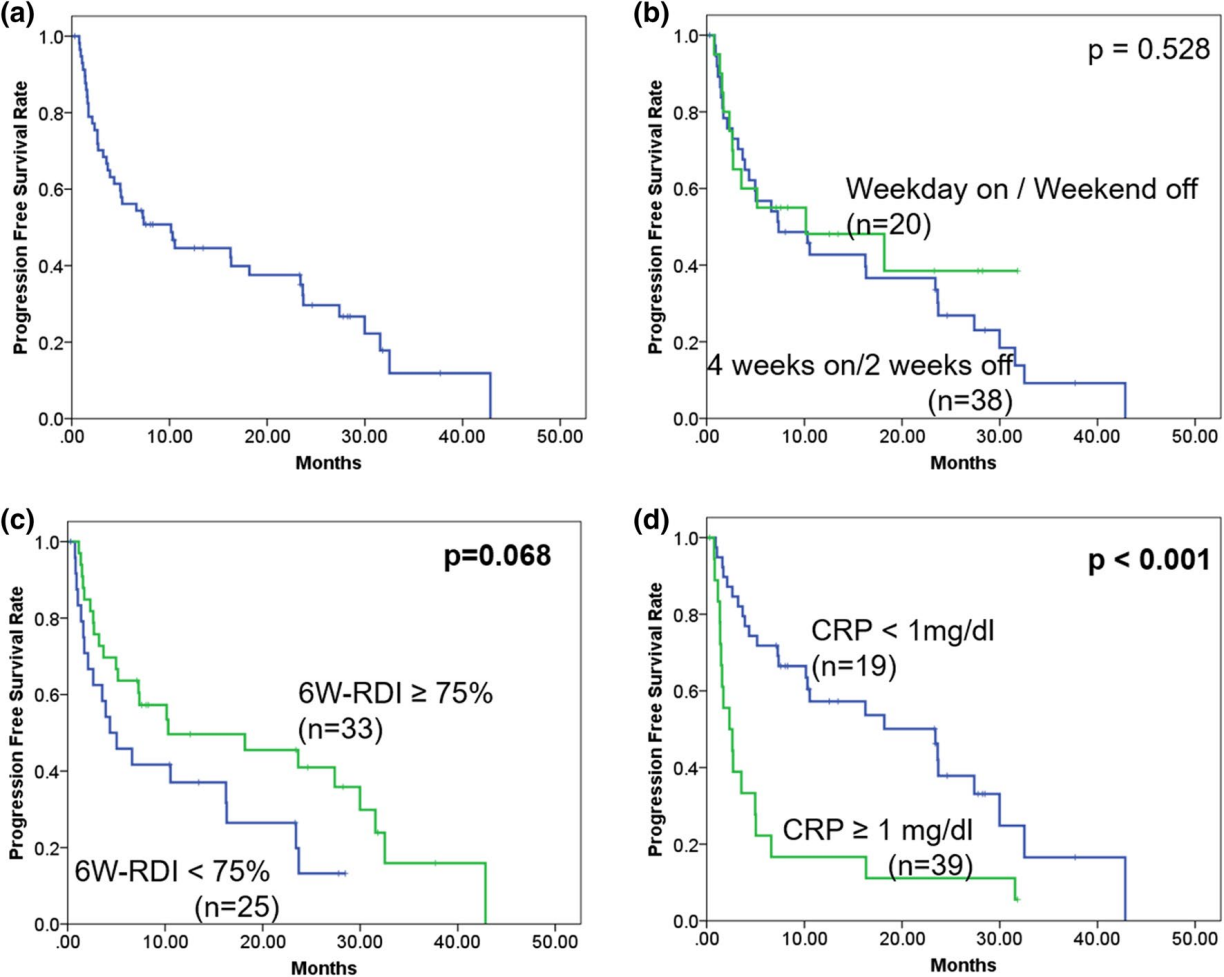
Progression-free survival curve of total cases (**a**) and stratified by administration schedule (**b**), relative dose intensity for initial 6 weeks (6W-RDI) (**c**) and C-reactive protein (CRP) (**d**). Statistical analysis was performed using log-rank test

The median PFS of total cases was 10.2 months (95% CI 4.1–16.3) (Fig. 3a). In subgroup analyses, there was no significant difference in PFS between “4 week on and 2 week off regimen” and “weekday-on and weekend-off regimen” (7.4 vs. 10.2 months, *p* = 0.528) (Fig. 3b). PFS of the patients with 75% and more 6W-RDI was better than that of patients with less than 75% 6W-RDI (10.3 vs. 4.3 months, *p* = 0.068) (Fig. 3c). PFS of patients with high CRP (1 mg/dL and more) was shorter than that of patients with low CRP (less than 1 mg/dL) (2.3 vs. 23.4 months, *p* < 0.001) (Fig. 3d).

In multivariate Cox regression analysis, low CRP (less than 1 mg/dL) [hazard ratio (HR) 3.014, 95% CI 1.587–5.725, *p* < 0.001] was identified as independent predictor of superior PFS time (Table 4).

**Table 4:** Result of univariate and multivariate analysis about the clinical features significantly correlated with progression-free survival. Statistical analysis performed using Cox regression analysis

	Univariate analysis			Multivariate analysis		
Clinical features	HR	95% CI	*p*	HR	95% CI	*p*
Age (median) (< 65 vs. ≥ 65)	1.382	0.736–2.597	0.314			
Gender (male vs. female)	0.716	0.299–1.713	0.453			
Initial sunitinib dose (50 mg vs. 37.5 mg)	0.960	0.513– 1.797	0.899			
Sunitinib administration schedule (Group I vs. Group II)	0.799	0.398–1.605	0.529			
Serum sodium concentration (≤ 137 mEq/L vs. ≥ 138 mEq/L)	0.654	0.344–1.244	0.195			
Estimated GFR (< 44 mL/min/1.73 m^2^ vs. ≥ 44 mL/min/1.73 m^2^)	1.348	0.725–2.509	0.346			
C-reactive protein (< 1 mg/dL vs. ≥ 1 mg/dL)	3.014	1.587–5.725	**0.001**	3.044	1.602–5.783	**0.001**
Relative dose intensity for initial 6 weeks (< 75% vs. ≥ 75%)	0.556	0.293–1.055	0.072	0.545	0.286–1.040	0.065

*HR* hazard ratio, *CI* confidence interval, *GFR* glomerular filtration rate

### Clinical features significantly correlated with discontinuation rate due to adverse events

**Fig. 4:**
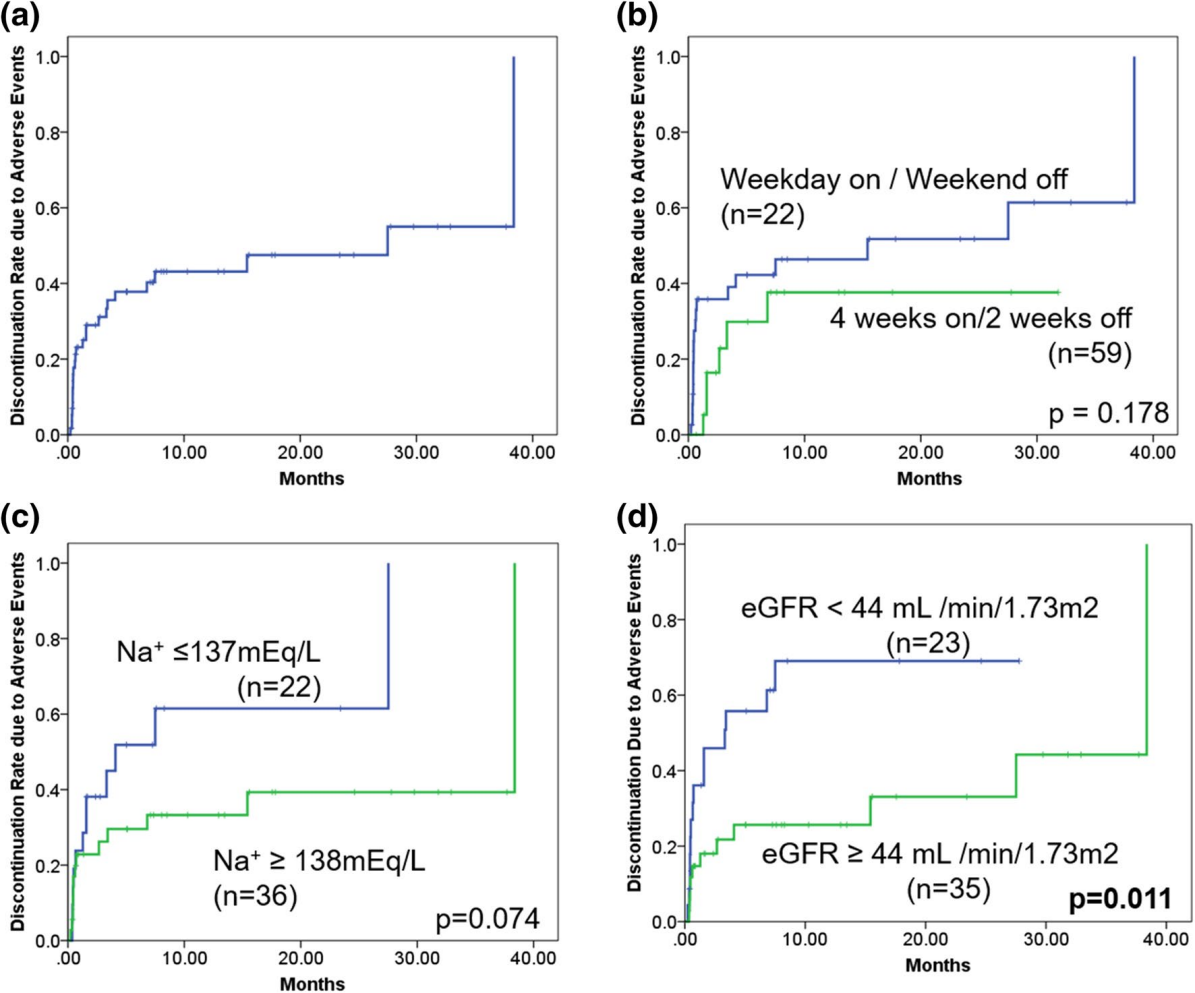
Discontinuation rate curve due to adverse events of total cases (**a**), stratified by administration schedule (**b**), serum sodium concentration (Na^+^) (**c**) and estimated glomerular filtration rate (eGFR) (**d**). Statistical analysis was performed using log-rank test

The median discontinuation time due to severe AEs of total cases was 27.5 months (95% CI 0.0–59.5) (Fig. 4a). In subgroup analyses, there was no significant difference regarding discontinuation time between the “4 weeks on and 2 weeks off regimen” group and the “weekday-on and weekend-off regimen” group (15.4 months vs. not reached, *p* = 0.178) (Fig. 4b). Discontinuation time for patients with hyponatremia (less than 138 mEq/L) was shorter than that of patients with normal sodium concentration (138 mEq/L and more) (4.1 months vs. 38.4 months, *p* = 0.074) (Fig. 4c). Discontinuation time of patients with impaired kidney function (less than 44 mL/min/1.73 m^2^) tended to be shorter than that of patients with normal kidney function (44 mL/min/1.73 m^2^ and more) (3.3 months vs. 38.4 months, *p* = 0.011) (Fig. 4d).

In multivariate Cox regression analysis, hyponatremia (less than 138 mEq/L) (HR 2.501, 95% CI 1.089–5.747, *p* = 0.031) and impaired kidney function (less than 44 mL/min/1.73 m^2^) (HR 3.216, 95% CI 1.395–7.412, *p* = 0.006) were identified as independent predictors of inferior discontinuation time (Table 5).

**Table 5:** Result of univariate and multivariate analysis about the clinical features significantly correlated with discontinuation rate due to adverse events. Statistical analysis was performed by Cox regression analysis

Clinical features	Univariate analysis			Multivariate analysis		
	HR	95% CI	*p*	HR	95% CI	*p*
Age (median) (< 65 vs. ≥ 65)	1.865	0.797–4.363	0.151			
Gender (male vs. female)	0.554	0.165–1.862	0.340			
Initial sunitinib dose (50 mg vs. 37.5 mg)	1.496	0.669–3.345	0.327			
Sunitinib administration schedule (Group I vs. Group II)	0.534	0.211–1.351	0.185			
Serum sodium concentration (≤ 137 mEq/L vs. ≥ 138 mEq/L)	2.064	0.915–4.656	0.081	2.501	1.089–5.747	**0.031**
Estimated GFR (< 44 mL/min/1.73 m^2^ vs. ≥ 44 mL/min/1.73 m^2^)	2.780	1.224–6.316	**0.015**	3.216	1.395–7.412	**0.006**
C-reactive protein (< 1 mg/dL vs. ≥ 1 mg/dL)	1.091	0.444–2.682	0.849			

*HR* hazard ratio, *CI* confidence interval, *GFR* glomerular filtration rate

## Discussions

Sunitinib is currently used as the first-line therapy for patients with metastatic or advanced RCC. Yet in addition to its efficacy as an anti-cancer agent, it often leads to serious side effects, requiring medication interruption or discontinuation in some patients [19]. Previous studies, including reports from our group, have shown that maintaining high RDI with molecular-targeted therapy improves the prognosis of RCC patients [6–8]. Thus, in an effort to reduce the occurrence rate of serious AEs while maintaining a high RDI, several groups have piloted new treatment regimens as alternatives to the conventional “4 weeks on and 2 weeks off regimen”, with some success. Here, we report the results of an alternative medication schedule, the “weekday-on and weekend-off regimen”, of sunitinib therapy for metastatic RCC patients.

As noted above, a number of alternative treatment regimens for sunitinib have been reported in the literature. Escudier et al. first reported phase II results of the “continuous one-daily dosing regimen” for cytokine-refractory patients. The median PFS time was 8.3 months, and the interruption and discontinuation rates were 65 and 78%, respectively. Severe adverse events included general fatigue (16%), diarrhea (12%), and hypertension (11%) [9]. Barrios et al. reported phase II results of the same regimen applied as the first-line treatment. Their median PFS time was 6.1 months, and interruption and discontinuation rate were 18% and 65%, respectively. Severe adverse events included hand-foot syndrome (13%), neutropenia (11%), and diarrhea (9%) [10]. Although the results of these studies appeared promising, a randomized phase II trial, which compared the efficacy between the “4 weeks on and 2 weeks off regimen” and “continuous one-daily dosing regimen”, proved that the conventional method was able to obtain better PFS than the alternative method [11]. Their results also indicated that the occurrence rate of severe AEs was almost the same between the two groups.

Subsequently, Najjar et al. reported the results of another alternative method, “2 weeks on and 1 week off regimen” [14]. They reported that severe AEs such as general fatigue or hand-foot syndrome were significantly reduced in this regimen as compared to the conventional method. Also, Bracarda et al. reported that the PFS time of the 208 patients whose medication methods were changed from conventional to the alternative method when severe AEs occurred was much better than the PFS of patients treated only by the conventional method (*n* = 211) or only by the alternative method (*n* = 41). The occurrence rate of AEs decreased in the alternative method group compared to conventional method group [12]. Recently, Lee et al. reported the results of a randomized phase II trial which compared the clinical utility of the “4 weeks on and 2 weeks off regimen” vs. the “2 weeks on and 1 week off regimen” [13]. In their RESTORE trial, they proved that the “2 weeks on and 1 week off regimen” demonstrated better failure free survival (FFS) than the “4 weeks on and 2 weeks off regimen” (median FFS 7.6 months vs. 6.0 months, *p* = 0.029) though there was no significant difference about PFS.

At the same time as these studies, we prospectively tried a “weekday-on and weekend-off regimen” as an alternative method of sunitinib therapy. Using historical controls for comparison, patients treated under the “weekday-on and weekend-off regimen” could achieve higher 6W-RDI than patients treated under the “4 weeks on and 2 weeks off regimen”. Also, the number of the patients who could get 75% and more 6W-RDI were statistically higher in the “weekday-on and weekend-off regimen” compared to those in the “4 weeks on and 2 weeks off regimen”.

In Japanese patients, severe thrombocytopenia occurred more often than in Western patients [5, 6]. In our cohort, severe thrombocytopenia was the most common AE. In terms of severe thrombocytopenia, the occurrence rate in “weekday-on and weekend-off regimen” was lower than in the “4 weeks on and 2 weeks off regimen”, although this difference did not achieve significance. This may explain why patients treated under the “weekday-on and weekend-off regimen” achieved higher 6W-RDI than those treated under the “4 weeks on and 2 weeks off regimen”.

Unfortunately, PFS for the “weekday-on and weekend-off regimen” was not improved compared to the “4 weeks on and 2 weeks off regimen” in both univariate and multivariate analysis. PFS of the patients treated with sunitinib in the general practice ranged from 5.7 to 9.4 months [5, 6], although PFS was much better in the clinical trials [3, 4, 20]. Total PFS in our cohort was similar to that of previous studies of general practice. CRP became statistically significant prognostic factor and higher 6W-RDI was tended to become significant prognostic factor in our cohort, as has been previously reported [6, 21]. Actually, 2-year PFS ratio of “weekday-on and weekend-off regimen” (58.4%) tended to be longer than that of “4 weeks on and 2 weeks off regimen” (31.9%) in 39 patients with lower CRP (*p* = 0.10) though that of “weekday-on and weekend-off regimen” (11.1%) was almost the same as that of “4 weeks on and 2 weeks off regimen” (11.1%) in 19 patients with higher CRP (data not shown). Collectively these results indicate that urologists and medical oncologists should seek alternative treatment regimens that maximize tolerated 6W-RDI for RCC patients with better prognostic factors such as lower CRP level.

In this study, discontinuation of sunitinib therapy was significantly correlated with the presence of hyponatremia and renal impairment. Chronic kidney disease was already reported to be significantly associated with toxicity-related treatment discontinuation [22, 23]. Hyponatremia is well-known to be correlated with poor prognosis in RCC patients treated with molecular-targeted therapy [24–26]. This is the first study to indicate that the presence of hyponatremia was significantly correlated with discontinuation of therapy in multivariate analysis. So, these patients should be treated more carefully along with modification of administration method because sunitinib therapy should be interrupted despite the therapeutic effect.

This study was subject to several limitations. This study was not a randomized control study, and the number of the patients enrolled was small. Nonetheless, we could achieve an improved 6W-RDI in our cohort, and this “weekday-on and weekend-off regimen” could be considered to manage patients to get prolonged PFS in general practice.

In conclusion, we report here the results of an alternative medication schedule, the “weekday-on and weekday-off regimen”, and suggest that this alternative could improve 6W-RDI in patients, a metric correlated with improved prognosis, and reduce the occurrence rate of severe AEs, although this difference did not achieve significance.
